# Institutional Needs

**Published:** 1913-03-29

**Authors:** 


### Institutional Needs.

### THE WELLBANK COOKER.

Not the least pressing of the many problems which confront the now very large population dwelling in flats is the question how to provide really hot meals without having to come home hours before meal time in order to prepare them. Now there is little doubt but that of all the various methods suggested towards solving this problem, the use of a Wellbank Boilerette goes farthest. Indeed, this Cooker is a success, not only as regards the actual cooking, but in the matter of convenience also. The Boilerette has already established itself on its merits, which include the following: It is self-acting and cannot spoil a good dinner, even though it be left for hours to itself; but far more important from a health point of view is the excellent way in which the food is cooked. Meat, poultry, and vegetables are virtually cooked in their own juices, and the salts and extracts become available in full instead of being lost. Moreover, it has a softening effect on "ancient" poultry or tough beef, which taste distinctly more tender when the Boilerette has finished with them. Unlike many ingenious devices for the small ménage, the Wellbank Cooker is economical in the matter of gas. This economy is easy to understand when the construction of the steam jacket with its spring safety valve is appreciated. This method seems an ideal application of the principle of water boiling under pressure as the source of an even, constant heat for cooking purposes. For the sick-room or as an adjunct to the small or private ward the Wellbank Boilerette can be confidently recommended.

